# Textile Design of an Intervertebral Disc Replacement Device from Silk Yarn

**DOI:** 10.3390/biomimetics8020152

**Published:** 2023-04-12

**Authors:** Michael Wöltje, Liesa Künzelmann, Basak Belgücan, Andreas S. Croft, Benjamin Voumard, Stefan Bracher, Philippe Zysset, Benjamin Gantenbein, Chokri Cherif, Dilbar Aibibu

**Affiliations:** 1 Institute of Textile Machinery and High-Performance Material Technology, Technische Universität Dresden, 01602 Dresden, Germany; 2 Tissue Engineering for Orthopaedic and Mechanobiology, Bone and Joint Program, Department for BioMedical Research (DBMR), Medical Faculty, University of Bern, 3008 Bern, Switzerland; 3 ARTORG Center for Biomedical Engineering Research, University of Bern, 3008 Bern, Switzerland; 4 Department of Orthopedic Surgery and Traumatology, Inselspital, University of Bern, 3010 Bern, Switzerland

**Keywords:** silk, intervertebral disc, annulus fibrosus, nucleus pulposus, embroidery, fiber-based additive manufacturing

## Abstract

Low back pain is often due to degeneration of the intervertebral discs (IVD). It is one of the most common age- and work-related problems in today’s society. Current treatments are not able to efficiently restore the full function of the IVD. Therefore, the aim of the present work was to reconstruct the two parts of the intervertebral disc—the annulus fibrosus (AF) and the nucleus pulposus (NP)—in such a way that the natural structural features were mimicked by a textile design. Silk was selected as the biomaterial for realization of a textile IVD because of its cytocompatibility, biodegradability, high strength, stiffness, and toughness, both in tension and compression. Therefore, an embroidered structure made of silk yarn was developed that reproduces the alternating fiber structure of +30° and −30° fiber orientation found in the AF and mimics its lamellar structure. The developed embroidered ribbons showed a tensile strength that corresponded to that of the natural AF. Fiber additive manufacturing with 1 mm silk staple fibers was used to replicate the fiber network of the NP and generate an open porous textile 3D structure that may serve as a reinforcement structure for the gel-like NP.

## 1. Introduction

Low back pain (LBP) is one of the most common diseases in today’s society; it affects up to 70% of the Western population at least once in their lifetime [1,2]. The high incidence of this condition has negative effects on both the quality of life of patients affected and on the whole economy. Hence, the socioeconomic burden associated with LBP is immense [3]. One of the primary causes of LBP is a degeneration of the intervertebral disc (IVD) caused by aging, impaired nutrient supply, or IVD trauma, caused by direct impact or indirectly initiated by fractures of the cartilaginous endplates.

The vast majority of back pain instances are functional and temporary. In order to treat degenerated IVD tissues, numerous surgical approaches and materials have already been investigated. These range from injectable biomaterials to IVD implants to restore the disc height [4,5]. Nevertheless, no fully satisfactory solution has yet been identified that results in the IVD’s reconstitution, thus leaving the patient with a disc that is not able to restore its initial height or has a risk of re-herniation. The current “gold standard” for surgical treatment of IVD-associated pain is to perform a spinal fusion of two or more adjacent vertebrae to stabilize segmental instability and to remove the damaged disc material; this is then filled with a metal cage containing bone grafts or substitutes. Although spinal fusion temporarily relieves the back pain, it seems that, at a later stage, the neighboring discs are more prone to accelerated degeneration, which again can become symptomatic (adjacent segment disease) [6,7,8]. Moreover, spinal fusion does not always succeed. An incomplete fusion can be found in up to 30% of all cases, which might result in the reappearance of back pain [9,10].

Therefore, implant strategies that replicate the natural anatomical structure of the IVD are required. Thus, researchers and clinicians are exploring tissue engineering strategies to improve IVD treatment. This requires complex structures made of different tissue types. They resemble the natural structure of tissues/organs, whereby the cells should be properly arranged in relation to each other. This is achieved via scaffolds, which should have a cohesive porous structure, appropriate pore size, and sufficient porosity to not only allow attachment, proliferation, and differentiation of cells, but also ensure distribution of bioactive substances and nutrient exchange during the development of new tissue [11]. From a structural perspective, an ideal scaffold should therefore have interconnected macropores with a size of 10 to 100 μm in order to facilitate cell infiltration and tissue formation, as well as a topography similar to the natural ECM, which can affect cell behavior, such as cell adhesion and gene expression [12,13,14,15,16]. Based on the concept of biomimetics: “there is no better model than nature to develop something new” [17]. To achieve this, the rational design of biomimetic scaffolds should be based on the natural structure and topography; this provides cells with a variety of physical, chemical, and biological cues that determine cell growth and function [18,19]. Therefore, to create an optimized cellular microenvironment conducive to the growth of 3D-structured tissue, biomimetic structures with controllable physical and mechanical properties, cell adhesion properties, and growth factor release kinetics are required [20]. For this reason, the design of the presented IVD replacement was such that the developed textile structure incorporates the idea of biomimetics.

To create a biomimetic IVD replica, it is important to understand the IVD’s anatomy. At the core of the IVD is a gelatinous tissue called nucleus pulposus (NP). It is a highly hydrated gel-like tissue with an abundance of distributed proteoglycans aggregating along hyaluronic acid chains [21]. The glycosaminoglycan side chains of these proteoglycans carry negative charges and are the prerequisite for the high water storage capacity of the NP [22,23] and thus, they provide the IVD with its high resistance to compressive stress. In addition, the NP is traversed by a fine collagen fiber network [24]. The NP is surrounded by the annulus fibrosus (AF). This tissue, unlike the NP, contains collagen fibers that make the tissue tensile and hold the softer and watery NP in place. The AF consists of 20–30 collagen fiber layers oriented in lamellar layers about 30–60° from vertical and alternating orientation of adjacent layers [25,26]. This fiber structure is responsible for the tensile strength of the AF [27,28,29]. Considering all this, it becomes evident that for the inner gel-like area of the NP, a porous textile structure is necessary; this serves as a reinforcing structure but leaves enough volume for the gel matrix to fill the entire area of the NP for pressure absorption. For the outer AF, a directional fiber structure is necessary; this can reversibly absorb the tensile force acting in the event of compression. Due to its outstanding textile-physical properties, biocompatibility, and slow degradation rate in vivo, silk was considered to be an eligible biomaterial to fulfill these requirements. Thus, the focus of this work was to develop a suitable silk-based textile structure to mimic the AF and NP. Fiber-additive manufacturing (FAM) was applied for NP assembly and embroidery for construction of the AF.

## 2. Materials and Methods

### 2.1. Silk Yarn

Two different silk yarns were used for the two different structures. For the embroidery of the AF, twisted schappe silk yarn of fineness 140/2 Nm (number metric) (Plauener Seidenweberei GmbH, Plauen, Germany) was used and Grège silk (40/42 den) was used for the NP. This yarn for the NP constructs consists of up to 10 threads and still contains sericin (Plauener Seidenweberei GmbH, Plauen, Germany).

### 2.2. Textile Design of AF

Based on the natural structure of the AF in the lumbar spine section (according to Frost et al. [30]), guideline values for the structure of the fibrous ring were determined. With a fiber orientation of +30 and −30 degrees, the structure of the single layer was created in SolidWorks as a contour line with the specified dimensions (length: 389.55 mm; height 10 mm) and the drawing was exported as a dxf-file. This was then used to generate a punch file for embroidery using the EPCwin software (ZSK, Stickmaschinen GmbH, Krefeld, Germany) and transferred to a format that could be embroidered. In order to minimize the number of joining points, a long ribbon was designed; this ribbon consisted of four layers of silk yarn, with each layer having an alternating fiber orientation of +30° or −30°. The ribbon was assembled on a ZSK Racer 1W embroidery machine (ZSK, Stickmaschinen GmbH, Krefeld, Germany). The distance of the yarn deposit was set at 0.7 mm and the silk yarn in each layer was embroidered on a double layer of the water soluble embroidery ground “Solvy Fabric No. 41825” (Gunold GmbH, Stockstadt, Germany). The same silk yarn was used for both the upper and the lower yarn. Together, these two yarn systems formed the layer structure for the individual layers, which together build up the annulus fibrosus ribbon. After embroidery, silk ribbons were separated from the embroidery ground by a two-step washing procedure. In the first step, three washing cycles were carried out. For this purpose, the specimens were soaked in lukewarm water in a washbasin for about 5 min each and then placed in a beaker filled with water. This procedure was repeated three times. The last rinse was conducted with distilled water in the beaker. In the second step, the pre-dissolved samples were placed in a water-permeable wire basket, which was then placed into a beaker and stirred at 300rpm for three days at 50°C in distilled water. Finally, the water was changed again and the patches were dried for one day at 60 °C. Then, the AF structure was assembled by winding the ribbon in the form of a snail shell. In this way, an AF structure consisting of 16 fiber layers was created. Finally, the rolled ribbon was joined at two positions (inside and outside) by manual sewing and the AF structure was fixed.

### 2.3. Regenerated Silk Fibroin Adhesive for FAM

For degumming, 2 g cocoon pieces of the silk moth *bombyx mori* were boiled under constant stirring in 1 L of 0.02 M Na_2_CO_3_ (Grüssing, Filsum, Germany) for 60 min and rinsed 3 times for 20 min [31]. Then, 1 g of degummed silk fibers were incubated in 10 mL (10%, *w*/*v*) of CaCl_2_/EtOH/H_2_O (molar ration 1/2/8) (*v*/*w*) at 65 °C for 3 h [32]. For desalting, the silk fibroin solution was dialyzed against distilled H_2_O using a Membra-Cel™ regenerated cellulose membrane with a molecular weight cut-off of 14,000 Dalton until the conductivity was below 10 µS. The regenerated silk fibroin solution was freeze-dried for 5 days using an Alpha 1-2 LDplus (Martin Christ Gefriertrocknungsanlagen, Osterode, Germany). For adhesive formulation, a 1% (*w*/*v*) solution of freeze-dried regenerated silk fibroin in 1,1,1,3,3,3-hexafluoroisopropanol (HFIP) was prepared.

### 2.4. Textile Design of NP

For generation of a textile NP structure with interconnected pores, the recently developed FAM technique was used as described in detail in [33]. In brief, in a preliminary step, the Grège silk yarn was cut into short fibers with a length of 1.0 mm. Before processing into 3D NP structures, the short fibers were degummed and dried again. The FAM process involves the following repeating steps: (i) deposition of a layer of anisotropic distributed silk fibers; (ii) spatial drop-by-drop dispensing of regenerated silk fibroin adhesive; (iii) deposition of a new fiber layer, which is connected to the previous one by silk fibers glued together at the points of adhesive deposition, thus simultaneously connecting the present layer with the previous one.

### 2.5. Scanning Electron Microscopy (SEM)

Silk textile AF and NP structure morphology was examined using SEM. Samples were sputter-coated with gold using a Cressington 108auto sputter coater (TESCAN, Dortmund, Germany) and imaged with a Quanta 250 FEG ESEM (Thermo Fisher Scientific, Waltham, MA, USA).

### 2.6. Tensile Testing of IVD Structures

Uniaxial tension testing was carried out for a group of 16 embroidered AF samples (8 dry, 8 wet). Test specimens were manufactured in the form of 4-layered ribbons, as described in Section 2.2. (Length: 125 mm, width: 10 mm).

Prior to mechanical testing, a μCT image of a water-soaked, pre-stretched silk strap was acquired at a spatial resolution of 11.4 μm and a field of view (FOV) of 35 mm (μCT 100, SCANCO Medical AG, Brüttisellen, Switzerland). X-ray tube voltage, current, and integration time were set to 45 kVp, 200 μA, and 300 ms, respectively, while using an aluminum filter with a thickness of 0.5 mm. Data averaging was set to 1 (one image acquired per slice). Image processing was performed using Python (Version 3.9.7). The scan was segmented using an Otsu threshold value. Apparent area was approximated as the cross-sectional area of the outer hull of the segmented image.

Mechanical tensile testing was performed using a multipurpose servo-hydraulic testing system (MTS 858 Mini Bionix, MTS Systems Corp., Eden Prairie, MN, USA) equipped with a linear voltage displacement transducer integrated into the actuator to measure displacement. Reaction force was measured using an s-beam load cell (RL20000A-100 SS, Rice Lake Weighing Systems, Rice Lake, WI, USA) with a load capacity of 450 N. Force and displacement signals were acquired at a frequency of 102.4 Hz and processed using MTS software (MTS FlexTest Station Manager Version 5.8B 5713, MTS System Corp., Eden Prairie, MN, USA).

**Figure 1:**
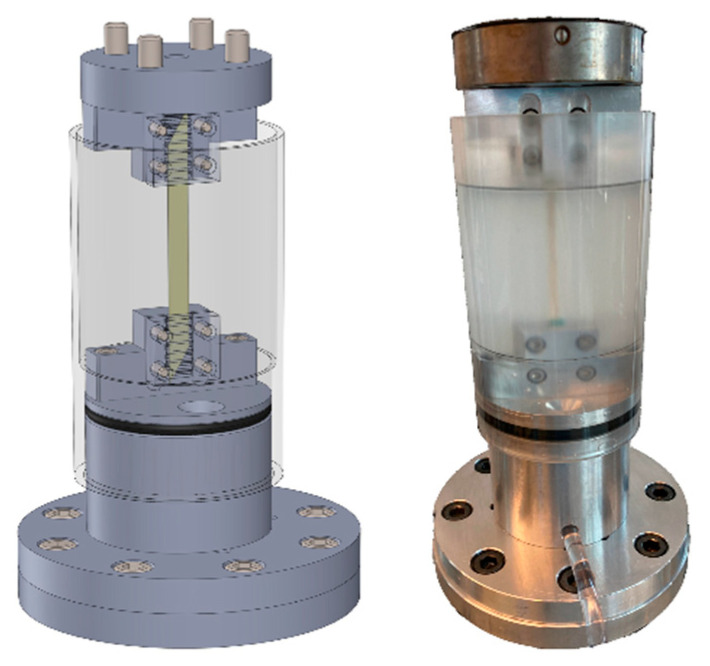
Mechanical testing setup showing CAD model (**left**) and actual configuration (**right**).

Eight samples were tested in dry conditions and eight in wet conditions. Dry state was reached after storage at a room temperature of 23 °C and a relative humidity of 45% for 5 days. Wet state was reached by fully immersing the samples in water for 20 min before testing and throughout the complete testing process. Silk straps were clamped using a custom-made device (Figure 1). Four M4 screws on each end were tightened with a torque of 3 Nm and the gauge length was adjusted to approximately 50 mm.

Zero strain state was defined by using a force-controlled mode up to a force of 12 N. Samples were cyclically loaded/unloaded in the elastic regime fourteen times up to a maximum force of 40 N, at a strain rate of 10 mm/min, using a displacement-controlled mode with subsequent linear monotonic loading up to failure using the same strain rate.

**Figure 2:**
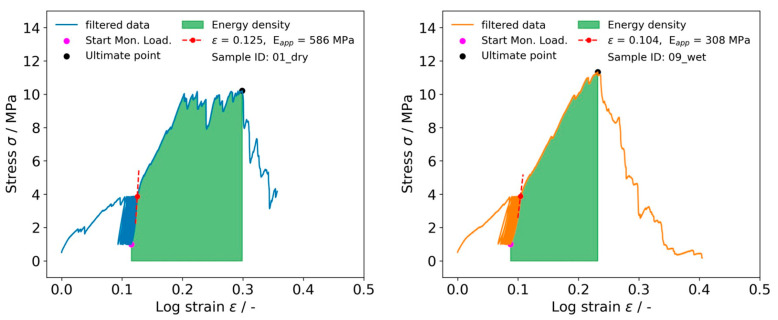
Stress/logarithmic strain curves for dry (**left**) and wet (**right**) samples indicating apparent modulus as slope at the beginning of the last unloading cycle as well as energy density, depicted as the area under the curve between the start of monotonic loading and ultimate point.

Apparent stress was calculated as

(1) $$\sigma =\frac{F}{A_{app}}$$

using the reaction force *F* and the mean apparent area extracted from the outer contour of the segmented μCT image. Ultimate stress was calculated by using the corresponding maximum force value. Logarithmic strain ε was defined as

(2) $$\varepsilon =log\left(1+\frac{\Delta l}{l_{0}}\right)$$

where Δ*l* and *l*_0_ depict the displacement and the original length, respectively. Ultimate logarithmic strain was evaluated at the point where maximum reaction force values occurred. A second-degree polynomial function was fitted on the stress/logarithmic strain data of the last unloading cycle of the preconditioning phase using a window width of 1/3 of the cycle length; apparent Young’s modulus E_m_ was calculated as the slope of the fitted curve at the start of unloading part (Figure 2, red dashed). Energy density U_T_ was calculated as the integral of the stress/logarithmic strain curve between the end of the preconditioning phase and the ultimate point (Figure 2, green).

Force-displacement signals were filtered by applying a digital filter forward and backward with a cutoff of 10 using Python and Scipy (version 1.7.3). Statistical analysis was conducted by performing two-sample *t*-tests using Python (version 3.9.7) and Scipy (version 1.7.3). Statistical significance was assigned to *p* ≤ 0.05. For each variable, the mean ± standard deviation was computed.

### 2.7. Determination of Porosity of NP-FAM Structures

Porosity and pore size distribution of the NP-FAM structures were measured using the liquid displacement method (Pore Size Meter PSM 165, TOPAS GmbH, Dresden, Germany), according to the manufacturer’s protocols. A value of 28.6 was used as the capillary constant. A perfluorocarbon (surface tension *σ* = 16 mN m^−1^) was employed as the test fluid, ensuring complete wetting of the NP structures without swelling. The size of the test specimens was 15 mm in diameter and 10 mm in height.

### 2.8. Statistical Evaluation

Statistical analysis was conducted using one-way analysis of variance (ANOVA) followed by Student’s *t*-test (independent, two sided). Differences were considered significant for *p* ≤ 0.05. All data are expressed as means ± standard deviation (SD).

## 3. Results

### 3.1. Textile Design of the Annulus Fibrosus

**Figure 3:**
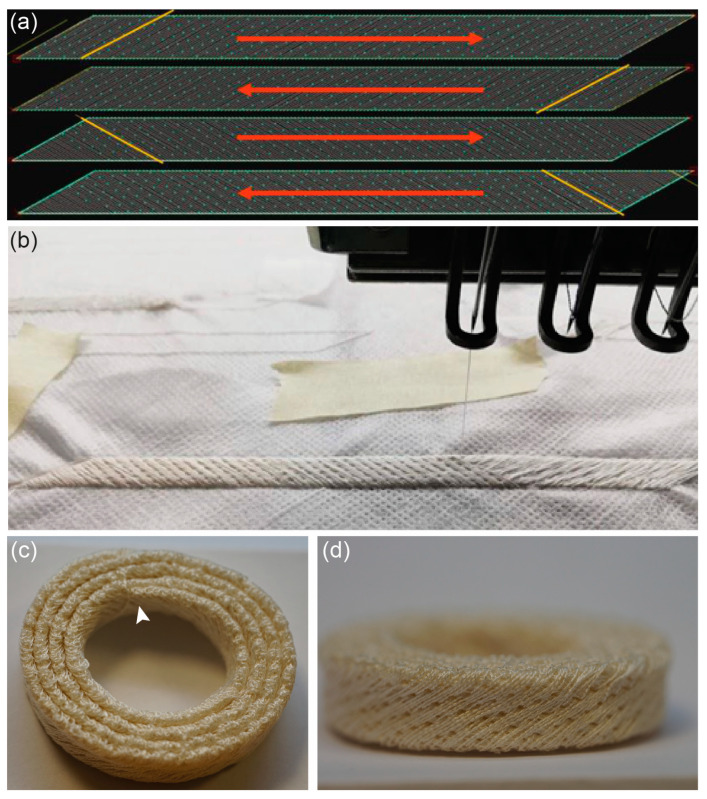
Embroidery of annulus fibrosus structures: (**a**) Representation of the variants of the pattern layers with stitch direction (yellow line) and embroidery direction from the start to the end (red arrow). (**b**) In-process picture of the production of an embroidered AF ribbon on the embroidery base. The alternating fiber deposition at the angle +30° and −30° can be seen clearly. (**c**) The embroidered AF structure was assembled and fixed by an inner (arrowhead) and outer seam. (**d**) Side view of the AF structure showing the uniform alternating yarn deposition that mimics the fiber orientation of the natural AF.

First, a textile structure was developed to mimic the AF as closely as possible to its natural state. Embroidery is a promising method for function, as it allows precise positioning of the yarn material. A two-thread system was used, consisting of upper and lower threads from silk yarn that cross at an adjustable point in the embroidery base. The lockstitch was used to form the lines and pattern filling. Unlike sewing, the position of the stitches to be inserted is determined specifically by a frame that moves in the X and Y directions, fixing the embroidery ground and moving according to the programmed pattern. Before embroidery, the pattern is created in CAD/CAM programs, which is technically called “punching”. As shown in Figure 3a, the four pattern layers with stitch direction (yellow line) and embroidery direction (red arrow) were developed using the EPCwin software. This software allows the creation of specific alternating fiber deposition patterns intended to mimic the natural AF fiber structure. After punching, the stitch data are transferred to the embroidery machine and the four individual layers are embroidered on top of each other to form a 4-layer ribbon. This process is shown in Figure 3b, where the upper thread of silk yarn and the embroidery head used to cross the yarn on the PVA embroidery ground can be seen. The alternating placement of the fibers, with a fiber orientation of +30° and −30°, is clearly visible. After removing the PVA embroidery ground by washing, the ribbon was rolled up into a snail shell shape in such a way that the beginning and the end were at the same height; this results in an AF layer of equal thickness being created over the entire perimeter of the IVD. Both ends were finally fixed inside and outside using one manual seam each (Figure 3c). The precise replication of the alternating fiber structure of the natural AF that can be achieved by the embroidery technique is evident in the side view of the textile AF (shown in Figure 3d); this figure also shows the uniform height of the textile AF structure over the entire extent of the IVD.

### 3.2. Textile Design of the Nucleus Pulposus

**Figure 4:**
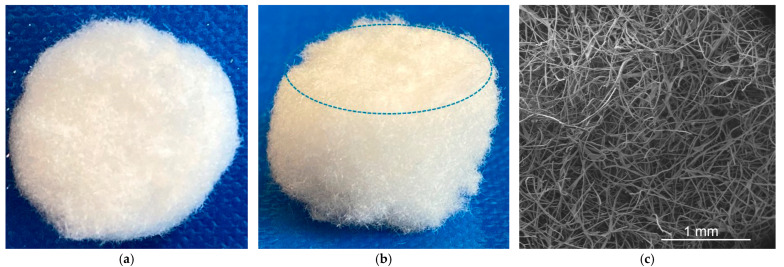
Textile FAM-made porous 3D nucleus pulposus structures: (**a**) Top view of the NP structure (diameter 15 mm), (**b**) Side view of the NP structure with the edge marked by a dashed circle (height 10 mm), and (**c**) SEM picture of the FAM NP structure.

The FAM process is a unique technology used for additive manufacturing of short fiber structures for regenerative medicine [33,34]. Similar to powder bed printing, this technology is a two-step process. First, a thin fiber bed made from silk yarn is deposited, and then a binder (in this case regenerated silk fibroin solution) is applied to bond the fibers together. The width of the fiber track depends on the fiber length used (1 mm). In the second process step, drops of the binder (freeze-dried regenerated silk fibroin dissolved in HFIP) are applied onto the fiber bed at defined deposition points by means of an air pressure-controlled extrusion nozzle (inner diameter: 200 µm) under computer control. Since the volatile solvent HFIP evaporates after deposition of the fibers, the short silk fibers are firmly bonded by regenerated silk fibroin. In this way, the porous textile NP was assembled layer by layer until the final height of 10 mm was reached (Figure 4).

**Figure 5:**
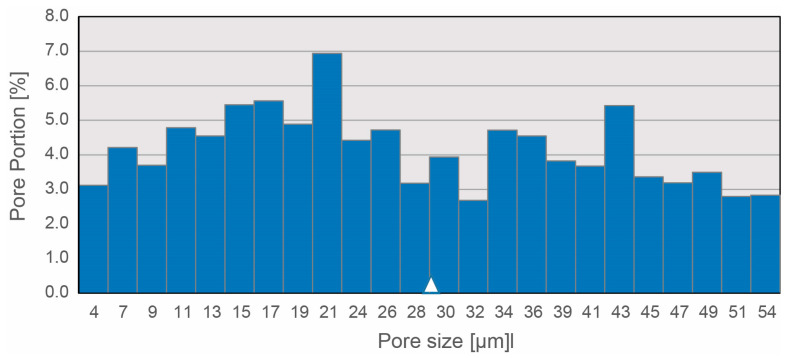
Pore size distribution in NP FAM constructs. The pore size is given in µm and the according pore portion in %. The mean pore size is shown by the white triangle (*n* = 3).

For characterization of the NP structures, the porosity and pore size distribution were quantified using the liquid displacement method. As shown in Figure 5, the constant pore size distribution ranges from 4 µm to 54 µm with an average pore size of 28.97 ± 1.77 µm and an average porosity of 94%. The FAM-NP structures were not investigated biomechanically because the pressure absorption of the IVD results only from the combination of the gel-like portion and the fiber portion of the NP. Therefore, a biomechanical characterization of the porous FAM-NP was not performed.

### 3.3. Characterization of Embroidered Annulus Fibrous Structures

**Figure 6:**
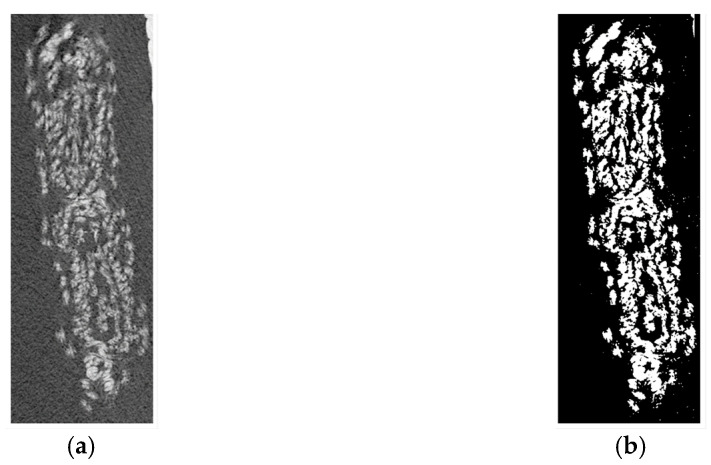
µCT analysis of embroidered textile AF specimen: (**a**) Scan and (**b**) binarized picture.

For the mechanical characterization of the embroidered AF structures, test specimens consisting of four layers with a length of 125 mm were fabricated. µCT analysis was used to estimate the apparent cross section of the silk embroidered AF structures in the prestressed state. Evaluation of the scan data (Figure 6a) and the corresponding binary images (Figure 6b) revealed an area fraction of 0.39 (4.21/10.77 mm^2^).

**Figure 7:**
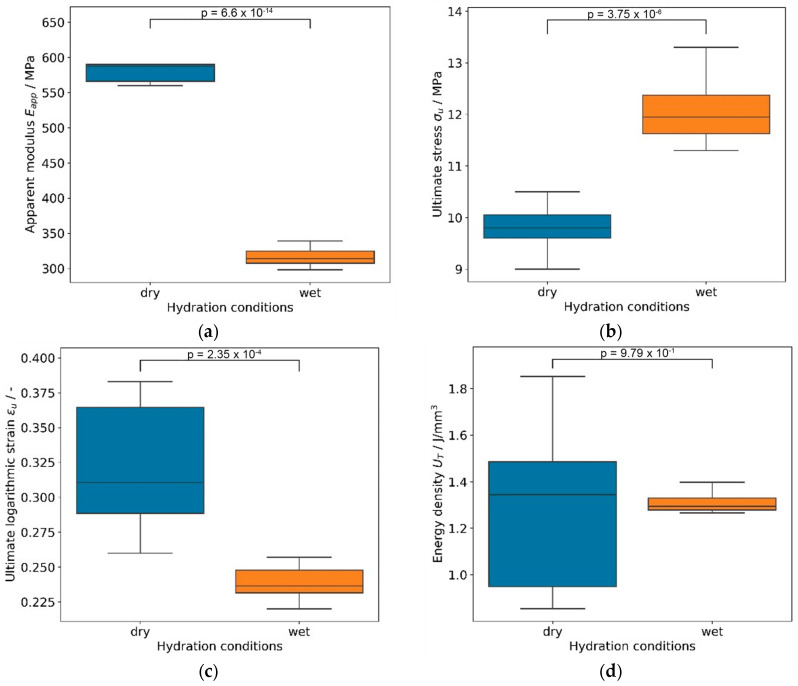
Box plots of mechanical variables showing significant differences (*p* ≤ 0.05) between wet and dry conditions for apparent modulus (**a**), ultimate stress (**b**), and ultimate logarithmic strain (**c**) but not for energy density (**d**).

As shown in Figure 7a, the apparent Young’s moduli $E_{app}$ were remarkably consistent within the groups but were significantly higher in dry versus wet conditions (584.3 ± 22.1 MPa and 317.9 ± 14.4 MPa, respectively). In contrast, the ultimate stresses $\sigma_{u}$ were significantly lower in dry versus wet conditions (9.8 ± 0.5 MPa and 12.2 ± 0.7 MPa, respectively) (Figure 7b). The corresponding ultimate strain $\varepsilon_{u}$ follows Young’s modulus and was significantly higher in dry versus wet conditions (0.32 ± 0.05 and 0.24 ± 0.01, respectively), as shown in Figure 7c. Combining the latter two effects, the energy densities to ultimate strain U_T_ did not show any significant difference between the two conditions (1.28 ± 0.35 J/mm^3^ and 1.31 ± 0.05 J/mm^3^) (Figure 7d). The variability of both ultimate strain and energy density appears also to be higher in dry conditions.

## 4. Discussion

The aim of this study was to develop a textile structure that mimics the oriented anisotropic multilamellar fiber network of the AF and the isotropic fiber network of the NP. Since the distinct oriented structure of the AF is particularly important for the mechanical functionality of the IVD and thus the spine, the mechanical properties of the textile structure to be developed should at least mimic the mechanical properties of its natural counterparts. Therefore, a new design of a silk fibroin implant was developed using embroidery, with fibers aligned at alternating angles in successive layers of a multilayered lamellar architecture for AF; short fibers were assembled by FAM to form an open porous 3D silk fibroin fiber network as a support structure for the gel-like NP.

Within the AF, the specific structural organization of collagen fibers results in nonlinear and highly anisotropic tensile behavior. Although the AF and its laminae are subjected to complex 3D loads during activities of daily living, an important type of loading on the AF is tensile. Tensile stresses on the AF are caused by radial forces resulting from compression of the gelatinous NP between the endplates of the vertebral bodies and the AF [35]. Therefore, uniaxial tensile stress measurement was used to characterize the embroidered textile AF structures in wet and dry conditions. The apparent area of the samples was estimated using μCT imaging of one sample and used for computation of apparent stress and apparent Young’s modulus. Furthermore, ultimate logarithmic strain and energy density were calculated and compared among dry and wet conditions by means of two-sample *t*-tests. Wet samples exhibited significantly lower apparent Young’s moduli, because the water acts as a plasticizer, promoting collective fiber rearrangement and recruitment in a parallel conformation, thus producing a higher ultimate stress. The dry samples did not exhibit this re-orientation; they were stiffer and failed more progressively with a higher ultimate strain but similar energy density. From a functional point of view, wet silk AF samples offered better recruitment of the load, higher strength, and more consistent mechanical performances.

The tensile strength of human AF is reported to be 4 to 10 MPa [36,37]. These values are reached by the wet embroidered AF structure presented here with 12.2 MPa, showing a tensile strength similar to human AF. In addition, the Young’s modulus of the embroidered AF structures is 317 MPa for wet samples. Several studies reported values for Young’s modulus of the annulus fibrosus of human IVDs within a range of 10 to 40 MPa [38,39,40,41,42], hence an order of magnitude lower than the moduli of the tested silk structures. It seems that the stiffness of silk fibers (~16 GPa [43,44]) and embroidered AF silk samples are one order of magnitude larger than the stiffnesses of the corresponding human tissue structures, which would be collagen fibers (~1 to 1.6 GPa [45,46]) and annulus fibrosus of IVD (~10–40 MPa). Here it should be noted that the two manual seams for fixing the ring structure represent a possible weak point regarding the load-bearing capacity of the textile AF structure. However, the spiral wound AF structure consists of eight additional layers without a seam; this contrasts with the tested four layer probes. Therefore, it may not be assumed that the force absorption capacity will be underestimated.

Previous studies using, for example, a multilamellar 3D scaffold with aligned electrospun nanofibers and FDM microfibers of PCL [47] or rolled tubular structures (six layers) of ±30° bilayer PCL:PLLA scaffolds [48] show a maximum tensile strength of only 2.6 MPa [47,48] and a Young’s modulus of 60 ± 16 MPa [48]. There are many other approaches to replicate the AF, but most of them only investigate compression for mechanical characterization (e.g., [49,50,51,52,53]). In addition, very promising approaches using 3D printing to rebuild IVD structures also achieved good mechanical strength in terms of compression [54,55,56,57]. However, not many data are available to compare tensile strength and Young’s modulus. In addition to the study presented here, several other approaches using silk as a biomaterial to replicate AF have been explored, but in most cases, regenerated silk has been used [58,59,60,61,62,63]. Only two other publications have used silk fibers to develop an AF structure. See et al. knitted a textile AF structure from raw silk fibers and placed it around a silicone core [64]. However, they did not provide experimental data on the textile properties of the knitted AF in terms of tensile strength and Young’s modulus. Another approach by Bhattacharjee et al. investigated a lamellar structure with crossing silk threads of tropical tasar non-mulberry silkworms *Antherea mylitta*. The results of an investigation of the tensile strength of the lamellar structures after cell seeding and dynamic cell culture showed a comparably high tensile strength (about 15 MPa), but the tensile strength of the cell-free AF structures was far below 5 MPa. A similar picture was found for the results on the Young’s modulus. The cell-free silk AF structures reached approx. 0.04 MPa. However, this increased to about 0.1 MPa after four weeks of cell cultivation. The observed increase of tensile properties may be attributed to the increased synthesis of extracellular matrix proteins by dynamic cell culture [65]. A similar result was obtained by Shamsah et al., but with electrospun bilayer scaffolds of PCL:PLLA,; they observed an increase in maximum tensile strength from 2.6 MPa without cells to about 8 MPa after cell colonization and a subsequent 14-day cell culture [48]. Furthermore, the mechanical properties of the AF could be further optimized by variation of the fiber orientation during embroidery, as shown for braiding of composite tubes [66,67].

Various biopolymers have already been investigated for mimicking NP tissue structures, mainly in the form of hydrogels such as collagen [68,69,70,71], alginate [72,73,74], chitosan [75,76,77], fibrin [59,60,78], hyaluronic acid [79,80], and silk fibroin [59,81,82,83,84,85,86]. However, the application of such hydrogels for tissue regeneration of NP structures is limited because the hydrogels have relatively weak mechanical strength that does not provide sufficient support to withstand the physical demands of the spine. In addition, although a hydrogel addresses the aspect of water retention, it completely ignores the fact that the NP is also composed of a fibrous structure, as shown by Tavakoli et al. [87] and reviewed by Cyril et al. [29].

Besides the IVD, many tissues are composed of fiber-reinforced hydrogel constructs or collagen fibers embedded in a gel matrix of elastin, e.g., in blood vessels [88], heart valve leaflets [89], skin, and tendons [90]. Therefore, the concept of fiber-reinforced hydrogel constructs is an effective tool to better mimic the structure and function of natural tissues. Collagen fibers determine the stiffness and strength in tension and mitigate the load-dependent response in compression, while the hydrogel-like elastin establishes a fluid pressure mechanism to support and distribute the compressive load [91,92]. In addition, fiber orientation, fiber architecture, porosity, matrix properties, and matrix-fiber bonding influence the mechanical properties of fiber-reinforced structures. Therefore, the mechanical properties of fiber-reinforced structures can be controlled by varying these parameters [93,94,95]. Thus, an open-porous textile structure was developed and additively manufactured to mimic the fiber structure of the NP.

For the development of textile NP structures using FAM, the porosity of the target structure was evaluated from publications on tissue engineering approaches to replicate the NP. This showed that, for example, porous cylindrical structures made of calcium polyphoshate, with an average pore size of 27 µm, were able to mimic natural tissue in vitro in terms of proteoglycan content and compression properties after colonization with NP cells [96]. Furthermore, using hydrogel scaffolds of glycerophosphate crosslinked chitosan and hyaluronic acid, Zhu et al. demonstrated that hydrogels with pore sizes in the range of 40–80 µm exhibited good mechanical properties and were found to be particularly suitable for promoting cell proliferation and differentiation of adipose-derived stem cells [97]. In addition, work with porous silk scaffolds generated by salt leaching showed that scaffolds with an interconnecting pore size of approximately 71 ± 26 µm exhibited a higher percentage of adherent cells, the largest growth curve, and accumulation of proteoglycans when compared to scaffolds with higher pore sizes [98]. For these reasons, the target porosity of textile NP structures should be in the range of 20 to 80 µm. Based on previous work on the simulation and design of textile FAM structures for tissue engineering [34,99], a fiber length of 1 mm was selected for development of the FAM structures. Thus, with an average pore size of about 30 µm, the textile FAM NP structures developed here were within the target size range.

However, since the pressure absorption of the IVD can only be achieved from the combination of the hydrogel and the fiber structure embedded in it, the textile NP structures were not investigated biomechanically. This is the subject of future studies, in which different hydrogels will be investigated in combination with the developed textile NP and AF structures to determine their suitability as IVD replacements.

In summary, the results presented here demonstrate the successful development of an IVD replacement device made entirely of textile building blocks that mimics the structure of both the AF and the NP. In addition, the developed embroidered AF structure exhibits a Young’s modulus and tensile strength similar to the natural AF. However, further investigations with hydrogel-filled NP structures surrounded by the embroidered AF textiles are necessary to show the suitability of such a textile IVD device for successful treatment of lower back pain.

## Data Availability

Not applicable.
